# Exploring the association between perceived male attitudes and female attitudes toward the discontinuation of Female Genital Mutilation/Cutting in Egypt

**DOI:** 10.3389/fsoc.2023.1183989

**Published:** 2023-07-13

**Authors:** Zita Zsabokorszky, Sarah Van de Velde, Kristien Michielsen, Nina Van Eekert

**Affiliations:** ^1^International Centre for Reproductive Health, Department of Public Health and Primary Care, Ghent University, Ghent, Belgium; ^2^Data Science Institute, I-Biostat, Hasselt University, Hasselt, Belgium; ^3^Centre for Population, Family and Health, Department of Sociology, University of Antwerp, Antwerp, Belgium; ^4^Research Foundation Flanders, Brussels, Belgium

**Keywords:** female genital cutting, perceived male attitudes, female attitudes, Egypt, FGM/C

## Abstract

**Introduction:**

There are 200 million girls and women alive worldwide that have undergone the practice of Female Genital Mutilation/Cutting (FGM/C) and 4 million girls are at risk of undergoing the practice each year. FGM/C provides no known health benefits, while puts a plethora of medical, psychological, and sexual health risks into perspective. One of the countries where the prevalence of FGM/C is the highest in the World, even though local authorities legally banned the practice in 2008, is Egypt. Within the Egyptian context, there are several complex socioeconomic, religious, and cultural drivers that influence the familial decision making of the daughters being cut. Female attitudes hold great significance in the process, because mothers and female family members are typically the prime decision makers at the daughter's circumcision. However, whilst FGM/C is often performed to enhance marriageability and address male preferences, in practicing communities there is little to no open communication between men and women about the practice, making women rely on their perceptions on FGM/C related expectations of men. Even though the connection between female and perceived male attitudes toward the discontinuation was established almost 20 years ago, since then to our knowledge little is known about the further characteristics of this association. Therefore, this study aims to explore the association between female and perceived male attitudes within families of a younger cohort and moreover attempts to provide a more layered picture of it within different levels of education.

**Methods:**

To explore the relation between female and perceived male attitudes toward the discontinuation of FGM/C we conducted a 3-step binary logistic regression model.

**Results:**

Our results show that women are significantly less likely to favor a continuation of FGM/C if they think men are disapproving of the practice, compared to women that think men want it to continue. The strength of this association partially varies between the different levels of education as it is less pronounced at the level of secondary education, compared to the reference group.

**Discussion:**

In alignment with previous findings in the literature, women were more likely to support the discontinuation of FGM/C if they believed that men want the practice to discontinue as well and vice versa. At a higher level of secondary education however this association is less pronounced. This result concludes that the role of perceived male attitudes should be an important factor associated with female ones and studied further, and underlines the importance of education in women empowerment.

## 1. Introduction

Female Genital Mutilation/Cutting (FGM/C) refers to the partial or total removal of the female external genitalia or other ways of harmfully altering the female reproductive organs for non-medical reasons (World Health Organization, 2023). Despite the health risks, there are still 200 million girls and women alive worldwide who have undergone the practice of FGM/C and 4 million girls at risk each year. The practice is mostly widespread in the Western, Eastern, and North-Eastern regions of Africa and some countries in the Middle East and Asia. Due to migration from these regions, FGM/C puts young girls and women at risk in Europe, Australia, and North America as well, making the practice of FGM/C a global health concern (World Health Organization, 2023).

Egypt has a special significance while analyzing the state of FGM/C in the World. Since the legal ban of 2008, which prohibits the practice of FGM/C in the country, the number of girls being cut, exposing them to various short and long-term health risks, is still strikingly high (Mostafa et al., 2020). While the overall prevalence of FGM/C in Egypt is 86%, regional prevalence vary between 62% and 93% (Egypt Family Health Survey (EFHS) from 2021). The prevalence is lower among girls between the age of 0–19 than among ever-married women, which shows the decline of the practice among younger birth cohorts. The proportion of circumcised girls (age 0–19) has declined from 35% to 13% since 2014, while the proportion of uncut girls that are expected to be circumcised declined from 56% to 27% (Central Agency for Public Mobilization Statistics, 2022). While the hazard ratio of FGM/C for younger birth cohorts is consistently declining since the 1980s (Van Rossem and Meekers, 2020), the magnitude of this trend was observed to be lower in Egypt compared to other African countries (Afifi, 2009). Results of a nationwide study furthermore suggest that 68% of male and 53% of female respondents still intended to circumcise their daughters in 2016 (El Feki et al., 2017). For this reason, exploring attitudes toward the discontinuation of the practice bears great importance.

In this regard, female attitudes are especially significant, as within the familial context, mothers and other female family members are often the prime decision makers of the daughter's circumcision (Suzuki and Meekers, 2008; Abdou et al., 2020). Whilst one of the main considerations behind such decisions is to enhance the daughter's chances for a favorable marriage by addressing male sexual preferences (Abdelshahid and Campbell, 2015), in communities where FGM/C is actively practiced, there is little to no open communication about the practice between sexes (Varol et al., 2015), making women rely on their perception of male preferences. Within this context, it is hard to decide how accurate these perceptions might be. Regardless of their accuracy however, a clear connection between female and perceived male attitudes, and between women's belief that men prefer the continuation of the practice and the intention to circumcise their daughter was established based on data from the early 2000s (Afifi and Von Bothmer, 2007; Suzuki and Meekers, 2008; El-Zanaty and Way, 2009). Since then, however, to our knowledge there is little amount of further evidence in the literature on the characteristics of this association. To address this gap, this study aims to explore if the association between female and perceived male attitudes still stands on comparable data from 2014 and explore its strength within different levels of education.

### 1.1. The main drivers of FGM/C in Egypt enhancing female acceptance toward the practice

The drivers that keep FGM/C alive in Egypt today are complex and deeply rooted in the local cultural context. The factors associated with female support of the practice can be divided into cultural (Almroth et al., 2001), social, and religious considerations (Alradie-Mohamed et al., 2020). The cultural roots of the practice originate in traditional views on female sexuality (Van Rossem et al., 2015, 2016). Today, especially in rural communities, undergoing FGM/C is often considered to be an important milestone for young girls coming of age, that also heavily determines their chances for a desirable marriage (Arafa et al., 2020). The reason for this is that traditional beliefs associate being cut with purity (Arafa et al., 2020), virginity (Mohammed et al., 2014), chastity and femininity (Abdelshahid and Campbell, 2015), and allows men to control the sexual relationship (Abdelshahid and Campbell, 2015). In these communities it is also associated with hygienic considerations and aesthetic preferences (Mohammed et al., 2014). Agreement with these beliefs (El-Zanaty and Way, 2009; Rasheed et al., 2011; Abdelshahid and Campbell, 2015; Wodon et al., 2017; Mostafa et al., 2020), and positive general attitudes toward the practice (Mohammed et al., 2018) are associated with the acceptance of it among women (Afifi and Von Bothmer, 2007). Undergoing the practice moreover enhances their qualities as wives and mothers and secures their social position within the community (Assaad, 1980; United Nations Childrens Fund Gupta, 2013).

Furthermore, FGM/C can be especially important for women from an economic point of view as well. The institutionalized gender inequalities within the Egyptian society rarely allow women to receive proper education and access the social and economic resources that are available for men. This creates great dependence on male family members (Yount et al., 2020), and later a favorable marriage (Yount, 2002). Consequently, the acceptance of traditional gender roles and discrimination, and favoring to marry at an early age are associated with the support of the practice among women (Wodon et al., 2017).

Lastly, the role of religion is also an important factor associated with the continuation of FGM/C in the country. Religious leaders have great power at shaping attitudes (Van Rossem et al., 2016) through the position and role they play within their communities (Arafa et al., 2020). While the Coptic Christian Church in Egypt decided to represent a united front against FGM/C and consciously targeted the elimination of the practice, the Islamic leaders are more fragmented in their opinion. This led to a faster pace of abandonment of the practice within the Christian communities compared to the ones of Muslim faith (Blaydes and Platas, 2020).

### 1.2. Male attitudes toward FGM/C in Egypt

Quantitative evidence on male attitudes toward the practice of FGM/C in Egypt is limited (Yousef et al., 2017), especially compared to the amount of evidence on male attitudes from neighboring countries (Ruiz et al., 2017). Existing local evidence however suggests that men are generally more in favor of the practice than women (Abdelshahid and Campbell, 2015). In 2016 around 70% of men, while only 50% of women supported the continuation of FGM/C in the country, which might be the result of the fact that preventive programs in the past decades mainly targeted women (El Feki et al., 2017). Similarly to women, acceptance of traditional sexual (El-Mouelhya et al., 2013; Abdelshahid and Campbell, 2015) and religious beliefs (Yousef et al., 2017), the wish to control the sexual relationship and prevent adultery (El-Mouelhya et al., 2013), and agreement with cultural and social traditions (Yousef et al., 2017) are also the main drivers of male acceptance of the practice. According to a nationwide survey, in 2017 74% of male respondents agreed that keeping up with the practice of FGM/C is important because of customs and traditions, 68% believed that it is required by religion, and 67% believed that FGM/C makes women less sexually demanding (El Feki et al., 2017). Complimenting these findings, by 2020 28% of Egyptian boys and men supported the discontinuation of FGM/C (UNFPA-UNICEF, 2020). Positive male attitudes toward the discontinuation are prominently associated with a higher level of education, deeper knowledge, and awareness of the negative health consequences of the practice (Varol et al., 2015; Yousef et al., 2017) and urban residence (Varol et al., 2015). Discussing the practice within familial setting is also becoming less of a taboo with time, especially within the urban context, which contributes to the wider acceptance of the abandonment of FGM/C (El-Mouelhya et al., 2013; United Nation's Children's Fund, 2023).

Qualitative research on male attitudes toward the practice reveals that male attitudes often appear to be ambivalent in nature (Almroth et al., 2001; El-Mouelhya et al., 2013; Varol et al., 2015; El Feki et al., 2017; Mohammed et al., 2018; Alradie-Mohamed et al., 2020). While men may acknowledge women's right to enjoy sexuality, which enhances positive attitudes toward the discontinuation of the practice, this was often overwritten by the wish to prevent adultery and maintain control over the sexual relationship (Almroth et al., 2001; Mohammed et al., 2014; Abdelshahid and Campbell, 2015), or worries about their own capability to sexually satisfy uncut women (El-Mouelhya et al., 2013). At the same time, social pressure and obligation for keeping up with the traditional values of the community (Varol et al., 2015), and the *silent culture* between sexes often prevent men from joining the discussion on FGM/C publicly in the practicing communities (Varol et al., 2015). Community pressure (Varol et al., 2015), and the fear of social rejection (Abdelshahid and Campbell, 2015) are often so strong, that even those men that worry about the negative health consequences of the practice prefer to marry circumcised women (Mohammed et al., 2014) and wish their daughters to be cut (Abdelshahid and Campbell, 2015).

### 1.3. Changing female attitudes toward FGM/C in Egypt

The prevalence of support of FGM/C's continuation among ever-married women has decreased from 62 to 58% between 2008 and 2014 based on the data of the Demographic Health Survey (DHS). The overall percentage of the practice however remained high within the Egyptian population (Alkhalaileh et al., 2018), as of today, 50% of women still support the continuation of the practice (El Feki et al., 2017).

Positive female attitudes toward the discontinuation of FGM/C before the legal ban (2008) were mostly associated with a higher level of education, urban residence and being uncut (Dalal et al., 2010; Alkhalaileh et al., 2018). Recent studies however identify various other factors, like enhanced knowledge on the negative health consequences of FGM/C (Afifi and Von Bothmer, 2007; El-Zanaty and Way, 2009; Dalal et al., 2010; Mitwaly et al., 2017), progressive views on sexuality, such as men prefer to marry uncut women, and that FGM/C can reduce sexual desire (Afifi and Von Bothmer, 2007), and higher level of female empowerment (Afifi, 2009; Modrek and Liu, 2013; Van Rossem et al., 2015).

Increased opposition also became more prominent across women with diverse background of education in the past decades (Van Rossem et al., 2016). For instance, in the 1990s only a higher level of education was associated with positive attitudes toward the discontinuation of FGM/C (Afifi and Von Bothmer, 2007; El-Zanaty and Way, 2009; Dalal et al., 2010; Van Rossem et al., 2016; Abdou et al., 2020). Lately however, the level of this connection seems to decrease, as from the early 2000s the opposition started to increase among lower educated woman as well (Van Rossem et al., 2015). This could be in alignment with the timely nature of this shift, as younger women across Egypt are also more likely to be uncut (Van Rossem and Meekers, 2020) and to support the discontinuation in general (Abdou et al., 2020). At the same time, the effect of specific educational programs on the negative health consequences of FGM/C targeting all segments of the Egyptian society also cannot be ignored (Afifi and Von Bothmer, 2007; Suzuki and Meekers, 2008; El-Zanaty and Way, 2009; Dalal et al., 2010; Rasheed et al., 2011; Mitwaly et al., 2017).

Similarly to education, the opposition also began to increase among women with lower status of wealth (El-Zanaty and Way, 2009) and more divers levels of socioeconomic background (Van Rossem et al., 2015). However, oftentimes mothers report to be held back from abandoning the practice out of fear of social exclusion, and because they don't see other means to secure their daughter's social position (Abdelshahid and Campbell, 2015) and a favorable marriage that they are oftentimes dependent on (Yount, 2002; Yount et al., 2020). This leads back to the pressure of marriageability (Wodon et al., 2017), and sufficing male sexual preferences (Abdelshahid and Campbell, 2015). Thus, exploring female attitudes toward the discontinuation of FGM/C could not be complete without including the role of men. The significance of their expectations translated by women, who try to become a more attractive partner for marriage through undergoing the practice (Abdelshahid and Campbell, 2015) is inevitable within the complex system of female attitudes.

Perceived male attitudes toward the discontinuation of FGM/C pose great importance, because even though open communication about the practice between men and women is generally not supported (Alradie-Mohamed et al., 2020), they are associated greatly with female ones (Afifi and Von Bothmer, 2007; Suzuki and Meekers, 2008; El-Zanaty and Way, 2009). The pressure coming from their husbands in favor of keeping up with the practice positively influences mother's decisions regarding their daughter's circumcision (Abdou et al., 2020), while the belief that men want the practice to discontinue is negatively associated with it (Afifi and Von Bothmer, 2007; Suzuki and Meekers, 2008; El-Zanaty and Way, 2009).

Since the connection between perceived male and female attitudes toward the discontinuation of FGM/C was established using data from the early 2000s (Afifi and Von Bothmer, 2007; Suzuki and Meekers, 2008; El-Zanaty and Way, 2009) and it hasn't been investigated since, there is no evidence on how this connection developed over time. For this reason, this study examines if the connection still stands on data from 2014. Moreover, it introduces control variables that could be connected to the changing nature of female attitudes discussed earlier. Namely, indicators of age, type of residence, level of education, religion, wealth index, employment, being cut, and the belief about the possible fatal consequence of FGM/C. The role of education is especially important within these factors, because recent evidence suggests that it is one of the most significant predictors of women's knowledge and attitudes toward FGM/C (Abdou et al., 2020). For this reason, current study aims to explore this connection within different levels of education and investigate if the association between perceived male and female attitudes is less pronounced with the higher level of education.

### 1.4. Hypotheses

Building upon the existing literature as discussed above we have formulated three hypotheses. In our first hypothesis we expect that women who indicated men support the practice of FGM/C will be more likely to support it themselves compared to women who think that men do not support it (H1). Closely connected to this assumption, we also expect that this difference holds when controlling to such sociodemographic and socioeconomic factors that partially explain it (H2). And finally, we expect that this difference will be more pronounced within lower educated women (H3).

## 2. Materials and methods

### 2.1. Sample

The Demographic Health Survey (DHS) Program, funded by the U.S. Agency for International Development (USAID) aims to assist institutions with collecting and analyzing health data from all over the world (ICF Demographic, 2018). This study analyses the Women's Questionnaire of the 2014 DHS Individual data set from Egypt, which was accessed and downloaded from the DHS's website after the positive evaluation of the research proposal. In the dataset, information was collected from a nationally representative sample of ever-married women between the age of 15–49 employing a multistage cluster sampling design. The standardized questionnaire used in this program was administered via face-to-face interviews. Respondents were asked about their socioeconomic and demographic characteristics, health, and specific questions about sexual and reproductive health, including questions on Female Genital Mutilation/Cutting. More information on the characteristics and findings of this data set can be found in an overview issued by the Egyptian Ministry of Health and Population (El-Zanaty, 2015).

### 2.2. Variables

#### 2.2.1. Female attitudes toward the discontinuation of FGM/C

To measure the dependent variable, female attitudes toward the discontinuation of FGM/C, this study used the question whether Female *Genital Circumcision should be continued or stopped*, because it gives clear indication of the respondent's affiliation. The variable originally had four values, *continued, stopped, don't know* and *depends*. In alignment with the statistical analysis, this variable was used representing two values, *continued*, and *stopped* (which was the reference category in the analysis). The *don't know* and *depends* answers (together making up around 11% of the responses) were coded as missing value, creating a dichotomous variable for the binary logistic regression model.

#### 2.2.2. Perceived male attitudes

To represent perceived male attitudes by our sample (which contained female respondents only), this study used the variable *Thinking that men want circumcision to continue or to stop*, which was introduced in the regression model as an independent variable. The original 3 values of the variable, *continued, stopped* and *don't know* were altered, coding the *don't know* answers as missing values to simplify the regression model.

#### 2.2.3. Control variables

The control variables applied in the regression model were chosen from a selection of demographic and social indicators already associated with female attitudes toward the discontinuation of FGM/C in the literature. A variable to represent knowledge on the negative health effects of FGM/C, which has proven to be associated with female attitudes was also included. Table 1 provides an overview on the control variables included in the logistic regression model.

**Table 1:** Overview of control variables included in the logistic regression model that explores the association between perceived male and female attitudes toward the discontinuation of FGM/C.

**Variable**	**Further explanation**
Age	In years.
Type of residence	This variable has two levels, *urban* and *rural*, which was the reference category in the analysis.
Level of education	The different levels of the variable are *no education, primary, secondary and higher* level. During the analysis, the level of *no education* served as a reference category.
Religion	This variable contains *Muslim, Christian* and *Other* value levels. Because the number of *other* religious affiliation was low (*n* = 17), it was coded as missing value to further develop the regression model. Next to the level *Christian*, the *Muslim* affiliation was used as a reference category.
Wealth index	This variable is divided into *Poorest, Poorer, Middle, Richer, and Richest* categories. As a reference category, the *Middle* level was used.
Employment	The two levels of the variable included *Respondent currently working (yes/no)*. The yes = working level was used as reference category.
Being circumcised	This variable was included with yes = circumcised as a reference category.
Thinking that circumcision can lead to a girl's death	The 3 levels of the variable were *Agree, Disagree* and *Don't know*. As a reference category *Agree (=thinking that circumcision can lead to a girl's death)* was used.

### 2.3. Statistical analysis

To test the 3 hypothesis introduced earlier, a 3 step binary logistic regression model was conducted. First, a binary logistic regression model was established to test the first hypothesis and explore whether there is an association between female attitudes and perceived male attitudes toward the discontinuation of FGM/C. Secondly, this regression model was expanded with the control variables (age, type of residence, level of education, religion, wealth index, employment, being cut, and thinking that circumcision can lead to a girl's death). Thirdly, an interaction term between perceived male attitudes and highest level of education was added to the model (Yount et al., 2020). All calculations were conducted in the Statistical Package for the Social Sciences (SPSS) downloaded for iOS.

## 3. Results

### 3.1. Characteristics of the sample

The original dataset contained 21,760 observations, of which 15,941 were included in the statistical analysis. The characteristics of the study sample stayed consistent with the original dataset after the exclusion of missing values and adjusting the variables to best fit the statistical analysis.

The study sample consists of ever-married women between the age of 15–49 (mean = 33 years). Almost 60% of these women had a rural residency, and 52% of them finished secondary education. The number of women that received no education at all was 23% of the sample, while 10% finished primary, and 14% finished higher education. Household wealth clusters (from poorest to richest) were represented in a balanced way within the sample, while 84% of the respondents was currently unemployed. A prominent majority, 96% of participants reported to be of Muslim faith. Coming to the specific questions regarding FGM/C, almost 90% of the sample was circumcised, and 64% of the respondents answered that FGM/C should continue. At the same time 64% thought that men want circumcision to continue, while 53% agreed with the statement that circumcision can lead to a girl's death.

### 3.2. Results of the 3-step regression model

Table 2, Model 1 shows that women are significantly less likely to favor a continuation of FGM/C if they think men are disapproving of the practice (OR: 0.01, *p* < 0.001). This effect holds when the results are adjusted by the controls variables, with only a small reduction in the effect size (Model 2; OR: 0.016, *p* < 0.001). Women with secondary (OR: 0.634, *p* < 0.001) or higher education (OR: 0.461, *p* < 0.001) also held less favorable attitudes toward the continuation of the practice compared to women with no education. Findings of this model additionally show that older women are significantly more likely to support the continuation of FGM/C (OR: 1.018, *p* < 0.001), while the type of residency (urban compared to rural) was not associated with a significant difference. At the same time women from the poorest households were significantly more likely to support the continuation of the practice (OR: 1.442, *p* < 0.001) compared to women from middle wealth households, while women from richer (OR: 0.686, *p* < 0.001) and the richest (OR: 0.520, *p* < 0.001) households were significantly more likely to support the discontinuation of the practice compared to the reference category. Women who currently did not work, compared to those who did were significantly more likely to support the continuation (OR: 0.825, *p* < 0.05). Looking at the role of religion, Christian women were significantly less likely to have positive attitudes toward the continuation (OR: 0.192, *p* < 0.001) compared to those of Muslim faith.

**Table 2:** Results of the 3-step logistic regression analysis exploring the association between perceived male and female attitudes toward the discontinuation of FGM/C (Model 1), adjusted for control variables (Model 2), and an interaction term exploring the association among different levels of education (Model 3).

	**Model 1**				**Model 2**				**Model 3**			
	**OR**	**95% C.I**.			**OR**	**95% C.I**.			**OR**	**95% C.I**.		
		**Lower**	**Upper**	**Sig**.		**Lower**	**Upper**	**Sig**.		**Lower**	**Upper**	**Sig**.
**Perceived male attitudes**												
Thinking that men want continue (ref.)												
Thinking that men want stop	0.010	0.009	0.012	^***^	0.016	0.014	0.018	^***^	0.010	0.007	0.014	^***^
**Education**								^***^				^***^
No education (ref.)												
Primary education					0.976	0.771	1.236		0.765	0.525	1.116	
Secondary education					0.634	0.534	0.752	^***^	0.453	0.351	0.586	^***^
Higher education					0.461	0.366	0.580	^***^	0.376	0.272	0.521	^***^
**Perceived male attitudes** ^*^education												^**^
Thinking that men want continue ^*^ no education (ref.)												
Thinking that men want stop ^*^ Primary education									1.539	0.944	2.511	
Thinking that men want stop ^*^ secondary education									1.873	1.344	2.612	^***^
Thinking that men want stop ^*^ higher education									1.402	0.917	2.144	
**Control-variables**												
Age					1.018	1.010	1.026	^***^	1.017	1.010	1.025	^***^
Type of residence												
Rural (ref.)												
Urban					1.095	0.905	1.324		1.095	0.905	1.324	
Wealth index								^***^				^***^
Middle (ref.)												
Poorest					1.442	1.176	1.769	^***^	1.446	1.178	1.776	^***^
Poorer					1.099	0.905	1.335		1.096	0.902	1.331	
Richer					0.686	0.556	0.847	^***^	0.685	0.555	0.845	^***^
Richest					0.520	0.405	0.667	^***^	0.518	0.404	0.664	^***^
Employment												
Yes (ref.)												
No					0.825	0.697	0.978	^*^	0.831	0.702	0.985	^*^
Religion												
Muslim (ref.)												
Christian					0.192	0.140	0.264	^***^	0.190	0.138	0.262	^***^
Respondent circumcised												
Yes (ref.)												
No					0.153	0.120	0.197	^***^	0.154	0.120	0.199	^***^
Thinking that circumcision can lead to a girl's death								^***^				^***^
Agree (ref.)												
Disagree					3.149	2.768	3.583	^***^	3.151	2.770	3.585	^***^
Don't know					2.143	1.695	2.709	^***^	2.154	1.703	2.724	^***^

^*^p < 0.05, ^**^p < 0.01, ^***^p < 0.001.

Coming to the questions relating to the practice itself, uncut women were significantly less likely to support the discontinuation than circumcised women (OR: 0.153, *p* < 0.001), while disagreement with the fact that FGM/C can lead to fatal consequences significantly raised the likelihood of positive attitudes toward the continuation of the practice (OR: 3.149, *p* < 0.001), compared to the opposite belief. Additionally, even those women who articulated an uncertain opinion were more likely to support the continuation of FGM/C than those who believed that it can lead to death (OR: 2.143, *p* < 0.001).

In the third model, the regression model was extended with the interaction term between perceived male attitudes and the mother's level of education to explore whether the researched difference in the previous models were greater within the various educational groups. However, this difference is not significant at the level of primary and higher education (comparing them to the reference category).

## 4. Discussion

### 4.1. Summary of the results

The result of the first logistic regression model confirms our first hypothesis and reveals that in alignment with previous findings from the early 2000s (Suzuki and Meekers, 2008): women were more likely to support the discontinuation of FGM/C if they believed that men want the practice to discontinue. This association was proven to prevail after applying our control variables, confirming our second hypothesis. Even though our results confirm that supporting attitudes toward the discontinuation of FGM/C are more common within younger (Abdou et al., 2020), uncircumcised (Dalal et al., 2010; Alkhalaileh et al., 2018), wealthier (El-Zanaty and Way, 2009), and Christian women (Modrek and Liu, 2013; Mohammed et al., 2014), our results suggests that women are still more likely to support the discontinuation of FGM/C if they believe that men support the discontinuation as well, regardless of their affiliation within these dimensions or age. This association also holds regardless of the belief that FGM/C can lead to death. This puts the importance of perceived male preferences into perspective, because even those women who know that FGM/C can lead to a girl's death are more likely to support the discontinuation of the practice if they believe that men want the practice to discontinue as well.

As we could see earlier, the role of education bears great importance regarding female knowledge and attitudes toward the practice (Abdou et al., 2020). While our results confirmed previous evidence according to which women with higher level of education are more likely to support the discontinuation of FGM/C (Afifi and Von Bothmer, 2007; El-Zanaty and Way, 2009; Dalal et al., 2010; Van Rossem et al., 2016; Abdou et al., 2020), the association between perceived male and female attitudes still held. However, when comparing the strength of this connection across different levels of education, results showed that it is less pronounced among women that received secondary education than among those that received none. This partially confirms our third hypothesis and underlines the importance of education once again. Our results not only suggest that receiving secondary education enhances the probability of supporting the discontinuation of FGM/C, but also conclude that it lowers the strength of the association between female and perceived male attitudes toward the discontinuation of FGM/C. Considering the local characteristics, higher educated women might be more willing to follow their own beliefs, because their more favorable social position could make them less dependent on their partners (Yount, 2002; Yount et al., 2020). It is still unclear however, why the strength of the connection between perceived male and female attitudes is only significantly lower at the level of secondary and not at higher level of education. A possible explanation could be is that it might be more common for higher educated women to think that men want the practice to discontinue than to think men want it to continue. This could be supported by the fact that 60% of respondents with higher education in the study sample believed that men want that practice to discontinue. However, further research is needed to explore this possibility.

### 4.2. Limitations

One of the main strengths of this study is the big and comprehensive sample size, provided by the DHS data set. This nation-wide sample of Egyptian women represents diverse segments of the society. However, there are important limitations that need to be addressed. Firstly, this study used the most recent available data, however it was collected almost a decade ago (2014). Secondly, the interview situation in which the data was collected might have pressured the respondents to provide socially conform answers, and the questions do not leave room to reflect on ambivalent feelings (Rasheed et al., 2011). It is also important to point out that that the “Don't know” and “Depends” answers that made up around 11% of the responses were coded as missing values in the analysis regarding the question if female circumcision should continue or to be stopped.

Regarding the control variables, there were no variables in the data set that would directly address women's social position and female empowerment. Even though such factors as education (Van Rossem et al., 2016), household wealth (Blaydes and Platas, 2020; Yount et al., 2020), and employment (Afifi, 2009) are good indicators for how accessible social and economic resources might be for women (Yount et al., 2020), they cannot fully grasp the added value of these factors. Furthermore, including variables to represent beliefs on traditional gender roles (Wodon et al., 2017), familial decision-making processes (Blaydes and Platas, 2020), exposure to messages on the negative health consequences of FGM/C (Afifi and Von Bothmer, 2007; El-Zanaty and Way, 2009; Dalal et al., 2010) and acceptance of gender-based inequalities and violence (Refaat et al., 2001) could have been valuable additions to the model.

Finally, this study focuses only on perceived male attitudes, whereas exploring the role of men could not be complete without investigating male attitudes toward the discontinuation of FGM/C. Possible discrepancies between male and perceived male attitudes by women may also occur, which was not possible for this study to account for due to data limitations. For this reason, it is impossible to know how accurate perceived male attitudes are in this study.

### 4.3. Conclusions and recommendations

There are many factors associated with female attitudes toward the discontinuation of FGM/C in Egypt today, and the results of this study support the evidence that the role of perceived male attitudes is one of them. The fact that women are more likely to support the discontinuation of FGM/C when they believe that men support it too leads to important recommendations.

Firstly, within the local context the role of men in the abandonment of FGM/C bears great importance. In those communities where FGM/C is actively practiced, such obstacles as social pressure and the *silent culture* between sexes make it almost impossible for men and women to communicate about the practice openly (Varol et al., 2015), while ambivalent, or positive male attitudes toward the discontinuation of the practice can hardly be articulated (Mohammed et al., 2014; Abdelshahid and Campbell, 2015; Varol et al., 2015). Thus, it is unclear how accurate perceived male attitudes toward the discontinuation of FGM/C are among women. For this reason, we recommend for future research to explore perceived male attitudes by women and compare them with direct male attitudes reported by men. We also recommend using both quantitative and qualitative research methods, which could not only facilitate to define the accuracy of perceived male attitudes by women but identify the main barriers of the abandonment of the practice.

Secondly, we recommend that educational and preventive programs address and involve men as well as women and invite them in an open dialogue on the discontinuation of the practice. This could be highly beneficial, because international experience shows that online campaigns, targeted media messages, forums and educational programs that incorporate and build on local characteristics can be highly successful at involving men in the neighboring countries of Egypt (Ruiz et al., 2016, 2017), countries where FGM/C is traditionally practiced (Small et al., 2020; UNFPA-UNICEF, 2020; Men End FGM, 2023) and in such countries as well, where FGM/C is only actively practiced by minority groups (Lien and Schultz, 2013; Vogt et al., 2017; Axelsson and Strid, 2020). Local evidence from Egypt also shows that preventive programs can successfully engage men (UNFPA-UNICEF, 2020; United Nation's Children's Fund, 2023) and that deeper knowledge and awareness on the negative health consequences of the practice are associated with positive male attitudes toward the discontinuation of FGM/C (El-Mouelhya et al., 2013; Varol et al., 2015; Yousef et al., 2017). Complementing this evidence, our results also underline the importance of male involvement in preventive programs, as what women perceive of their attitudes toward the discontinuation of FGM/C potentially shapes their own attitudes. Building on this evidence and involve men in preventive programs as well as women could create an active discussion between sexes and allow men and women to express their support toward the discontinuation of FGM/C more freely. Engaging men in active participation could be a very powerful tool to achieve the eradication of the practice by 2030 in Egypt.

## Data availability statement

Publicly available datasets were analyzed in this study. This data can be found here: Demographic Health Survey, Egypt, 2014, Standard DHS, DHS-VI, https://dhsprogram.com/data/available-datasets.cfm.

## Ethics statement

Ethical approval was not required for the study involving human data in accordance with the local legislation and institutional requirements. The analysis was carried out using the existing data obtained from the Women's Questionnaire of the 2014 DHS Individual dataset from Egypt.

## Author contributions

ZZ carried out thesis work at the Inter-University Advanced Master's Program of Global Health (Ghent University, Belgium). ZZ took the lead, while NV, SV, and KM guided her throughout the process. Several discussions were carried out about the research aim, methodology, and interpretation of the results. ZZ carried out the literature review, participated in the study design, accessed, and processed the data, contributed to the statistical analysis, and drafted the manuscript. KM coordinated the research project, participated in the study design, and was consulted about the results of the study. SV participated in the study design, data management, interpretation of the results, carried out the statistical analysis, and provided supervision of the manuscript. NV participated in the study design, the data process, statistical analysis, the interpretation of the results, and provided constant supervision and support at drafting the manuscript. All authors have read and approved the final document.
