# Corrigendum: Exploring the association between perceived male attitudes and female attitudes toward the discontinuation of Female Genital Mutilation/Cutting in Egypt

**DOI:** 10.3389/fsoc.2023.1353912

**Published:** 2024-01-04

**Authors:** Zita Zsabokorszky, Sarah Van de Velde, Kristien Michielsen, Nina Van Eekert

**Affiliations:** ^1^International Centre for Reproductive Health, Department of Public Health and Primary Care, Ghent University, Ghent, Belgium; ^2^Data Science Institute, I-Biostat, Hasselt University, Hasselt, Belgium; ^3^Centre for Population, Family and Health, Department of Sociology, University of Antwerp, Antwerp, Belgium; ^4^Research Foundation Flanders, Brussels, Belgium

**Keywords:** female genital cutting, perceived male attitudes, female attitudes, Egypt, FGM/C

In the published article, there was an error regarding the affiliations for Nina Van Eekert. As well as having affiliation 3, they should also have 4. Research Foundation Flanders, Brussels, Belgium.

In the published article, the Funding statement was missing. The correct Funding statement appears below.

